# Parental Involvement and Stress in Children’s Quality of Life: A Longitudinal Study with Portuguese Parents during the COVID-19 Pandemic Period

**DOI:** 10.3390/children11040440

**Published:** 2024-04-06

**Authors:** Helena Mocho, Cátia Martins, Rita dos Santos, Cristina Nunes

**Affiliations:** Psychology Research Centre (CIP), University of Algarve, Campus of Gambelas, 8005-139 Faro, Portugal; hsalcaparra@ualg.pt (H.M.); csmartins@ualg.pt (C.M.); rasantos@ualg.pt (R.d.S.)

**Keywords:** parental involvement, parental stress, children quality of life, COVID-19

## Abstract

Parental involvement (PI) has particular relevance on children’s academic adjustment and on children’s general quality of life (QoL). QoL can be influenced by parental stress, specifically the stress suffered during the COVID-19 pandemic. Thus, this study aimed to analyze the differences during the pandemic period (before, after and with no lockdown), comparing these constructs with parental educational level to provide predictors of their children’s quality of life. Data collection was performed with a non-probability convenience sampling procedure. It was composed of 129 parents, mainly women (71.8%), with children aged between 6 and 12 years. The family’s sociodemographic characteristics were assessed, as well as the PI, parental stress, and children’s QoL. The comparison between the three times revealed an increase in the children’s QoL, but no differences were found in PI. Based on the parental educational level, as defined by academic qualifications and split into superior and non-superior levels, it is possible conclude that engagement in school activities and parents’ meetings increased in the parents with superior education levels and decreased in the parents with lower education levels. This study concluded that despite this difficult and uncertain pandemic period, these parents were able to maintain important aspects of their children’s lives.

## 1. Introduction

Parental involvement (PI) is a “multifaceted and multidimensional phenomenon” [1] (p. 34), that has particular relevance in children’s academic adjustment [2,3] and positive development [4,5,6,7]. Some authors define PI in a more general way, focusing on the investment and cooperation of parents [1], but others focus more on specific features and types of involvement, such as the type of strategies used (e.g., home-based strategies or school-based strategies) [3]. PI can be defined as parental participation in children’s educational processes and experiences [8,9], including several activities that take place within the family (e.g., listening to children read or supervising homework), and school contexts (e.g., attending education seminars or parent-teacher meetings) [10]. It comprises the domains of the children’s social, emotional, and adjustment process [1]. Therefore, it has been widely recognized as a determinant of children’s learning, academic and social performance, and engagement [3,10,11]. PI can contribute to children’s well-being and quality of life [12]. Many studies have also pointed to it as a protective factor [1].

Regarding PI predictors, studies have pointed to characteristics of the child, family, and school, the child’s school experience, and the degree of parental satisfaction with school services [11]. PI has several benefits for the family and child domains, namely, a better relationship between teachers and parents, a more satisfactory school climate, higher school attendance, improvements in children’s attitudes, behaviors, and mental health, and greater parental confidence, satisfaction, and interest in their own education [13]. Ultimately, more parental involvement in a child’s education can also be reflected in better outcomes at school [1,3,14]. Although some authors have emphasized that PI decreases with children’s age, e.g., [15], a systematic review showed that it does not decline, but it does change over time, and the direct involvement of parents in orientations or other practices of direct support on school tasks are more effective in the initial stages of schooling [3]. 

Unlike children, for whom the PI in school activities is very important, for adolescents the PI in at home activities is one of the most relevant features [16,17]. As they get older, children try to assert their independence and are not so enthusiastic about overt parental involvement at school [17]. Instead, adolescents usually prefer more covert forms of PI, such as getting advice from their parents on various school-related activities [16,17]. 

Many family factors, such as the parental education level, can influence a child’s learning achievement [18]. The parents’ level of education has a relevant impact on the child’s academic development [19,20]. Differences between fathers and mothers were also found, and the literature emphasizes the relevance of a father’s involvement. Paternal involvement can have positive consequences for a child’s development, not only through the time spent and activities developed with the child, but also through the education and support provided, assistance with decision-making, and monitoring the child [21,22]. Direct, indirect, or mediated effects of PI on children’s outcomes are identified in this paper, as well as the influences of motherhood. Positive child behaviors, in turn, also generate greater parental involvement [22].

School contexts can be challenging and highly demanding for children, and PI can be a protective factor. These can play a central role in the development of vulnerability to stress, as children who grow up in appropriate contexts tend to be more resilient and overcome these experiences more efficiently [23]. The family can be a facilitator in the development of resilience. On the other hand, it may enhance vulnerability, either by its characteristics and family relationships or by the presence or absence of illness, types of resources, and support. Events outside the family can also disrupt its balanced and healthy functioning. So although parents can be moderators between stressors and the child, they can also experience stress and transfer it to their children, becoming stressors themselves [24].

The presence of high levels of parental stress (PS), occasional or chronic, can cause changes and contradictions in the parental educational style, culminating in coercive and poor parenting practices and physical abuse, drop and neglect practices, or inadequate educational practices, together with a strong lack of interest in monitoring the children’s lives [24]. Moreover, it can enhance parents’ insensitivity to their children’s characteristics, maximizing their negative behaviors in daily life, fostering rigid and automatic decision-making, and limiting parents’ focus on the consequences of their educational actions toward children [25]. Consequently, children are conditioned by these tensions and may develop socioemotional and behavioral or school problems. This causes negative perceptions of the child by the parents, increasing the likelihood of maltreatment of the child [25]. Nevertheless, in Southern European countries such as Portugal, there is scarce empirical data on the relationship between PS and negative life events [26] like the COVID-19 pandemic.

PS is indirectly related to children’s cognitive development and prosocial behavior through parental responsiveness. Paternal and maternal stress are both directly associated with behavior problems [27], and maternal PS usually is associated with negative outcomes for children [28]. However, PS is known to influence other parents’ parenting behavior, supported by the mutual effect of the parenting context [28]. According to the literature, mothers exhibit higher levels of PS compared to fathers. However, PS may be a determinant of parent-child interactions and child outcomes [27]. 

Between 2020 and 2021, the world witnessed a general lockdown, in which children and parents experienced a change in their main routines. Suddenly, families faced several challenges in their leading life roles (e.g., family, work, school, and social) [29]. During the COVID-19 pandemic, most governments around the world implemented strict domestic quarantine policies to control the spread of the disease [30]. Abrupt and prolonged school closures, disease containment measures, and economic impacts during the COVID-19 pandemic had serious implications for all aspects of child development, including physical, psychosocial, cognitive, and mental health, as well as on family relationships [31]. 

Children can be more sensitive to changes in routine, therefore parents tried to reduce children’s stress levels during lockdown [32]. During these periods, especially in the beginning of the pandemic, children experienced high levels of stress, caused mainly by fear [33], anxiety, and depression (the most common symptoms in children during the COVID-19 lockdown), but also loneliness, psychological distress, anger, irritability, boredom, and eating disorders [34]. These abrupt changes (deprivation of interaction with family and peers, school closures, changes in the way they studied, lack of outdoor activity, and irregular eating and sleeping habits) were likely to disrupt the children’s usual lifestyle and increased feelings like monotony, distress, impatience, boredom, and various neuropsychiatric manifestations [35]. The interaction between lifestyle challenges and the psychosocial stress caused by lockdown can exacerbate the adverse effects on a child’s physical and mental health, which can lead to a vicious circle [32]. All of us saw this complex interaction between psychosocial stress and the pandemic, caused by the lockdown and immediate changes at home, at school, and in the lifestyles of children [35].

Within this scenario, parents were asked to view themselves as facilitators, to actively participate in their children’s teaching-learning process, to maintain and promote their children’s school motivation and academic adjustment, and to minimize the consequences of the deprivation of interactions with peers and the rest of their support network, ultimately striving to maintain family mental health [36,37]. Studies developed during this period showed that in some circumstances, when they had to provide support for their children, for example regarding school tasks, parents tended to experience high levels of stress (e.g., [38,39,40,41]). Hence, the way in which parents perceived the lockdown could change their relationships with their children, which played a critical role in determining their stress levels. Those who saw lockdown as an opportunity to share more time with their children expressed lower levels of parental stress [42].

With respect to PI, around the world, several investigations were developed to study this phenomenon during the COVID-19 pandemic (e.g., [39,40,43,44,45]). Atypical circumstances such as a lockdown can challenge parenting skills and, consequently, increase the level of parental stress [40], since parents are unable to respond adequately to the needs of their children [46]. 

Regarding PI and children’s age, although parents have become more engaged in their children’s education [3,47], some literature has pointed out that parents tend to be more engaged when their children are in preschool or elementary school and tend to decrease their involvement as their children advance in schooling (e.g., [17,48]). Other studies have shown that parents do not decrease their involvement, they change it [47] due to (among other aspects) a development perspective, since children modify their interests and move to a peer-group and outside-family group focus [17,48,49]. Although these studies are pre-COVID-19, it is reasonable to assume that during this period, parents’ engagement in school tasks and learning management and motivation suffer some restructuring and even increase [40].

Family is one of the most important contexts of a child’s life, so parental engagement is likely to influence a child’s Quality of Life (QoL). The QoL of children and adolescents is related to an individual’s self-perceived health, including physical, emotional, social, and school well-being [50]. It is defined as a multidimensional, holistic, and subjective construct that changes over time [51,52,53]. QoL constitutes an important element of mental health [54]. The rate of children and adolescents with low QoL was notably higher during the lockdown [52,55] and various psychosomatic symptoms such as irritability, sleep problems, headaches, low energy, and stomach pains were observed, while the risk of mental health issues increased [55]. Also, contextual factors were identified (e.g., lower physical activity and sports, habitational conditions such as overcrowding, and changes in their social networks [56]).

Thus, given the pandemic period we were experiencing, the QoL of children were basic points to be considered, since a child’s positive and healthy development, as well as possible changes in behavior, beliefs, and attitudes, are a consequence of the interaction and influence of multiple contextual variables in the child’s life [57]. Considering the relevance of PI and PS in a child’s QoL, we decided to analyze the differences during a pan-demic period (i.e., before, after, and with no lockdown). The following research questions were developed:

**RQ1:**  *Do the levels of PI, PS, and children’s QoL vary before, after, and with no lockdown?*

**RQ2:**  *Are there differences in PI, PS, and children’s QoL before, after, and with no lockdown, according to parental levels of education (i.e., superior, and non-superior)?*

**RQ3:**  *What are the best predictors of children’s QoL before, after, and with no lockdown?*

Based on the evidence, we hypothesized that: (H_1_) PI and PS would increase from before, after, and with no lockdown; (H_2_) Children’s QoL will decrease from before to after a lockdown; (H_3_) The parents’ level of education is the most relevant positive predictor of PI and QoL, and PS is the most relevant negative predictor; (H_4_) PI is a relevant predictor of QoL during the pandemic period (before and in the end of the lockdown).

## 2. Materials and Methods

### 2.1. Sample

Data collection was conducted at three times: the first time in January of 2021 (beginning of a lockdown; T1); the second time in March of 2021 (post-lockdown; T2), and the third time at the end of the school year, June of 2021 (no lockdown; T3). 

The total sample (T1) consisted of 129 parents, mainly women (71.8%), with children aged between 6 and 12 years (*M* = 8.57, *SD* = 2.60). The mothers’ ages ranged from 29 to 50 (*M* = 39.41, *SD* = 4.11), and fathers’ ages from 30 to 55 (*M* = 41.38, *SD* = 4.86). In the T2 collection, 109 parents participated (84.5%), mainly women (69.7%), in which mothers were aged between 30 and 50 (*M* = 39.47, *SD* = 4.05) and fathers between 30 and 55 (*M* = 41.38, *SD* = 4.94). Finally, in T3, 88 parents participated (68.2%), mainly women (68.2%), with ages ranging from 30 to 50 (*M* = 39.40, *SD* = 3.88) and fathers from 32 to 54 (*M* = 41.33, *SD* = 4.68). All of these Portuguese families live in the south of Portugal. 

Regarding the families’ composition, 93% were biparental and had between 1 and 4 children (*M* = 1.74, *SD* = 0.64). In total, 225 children were included in the households, aged between 0 and 17 years old; 140 children were between 6 and 12 years old (*M* = 8.98, *SD* = 1.81) and attended either elementary (63.6%) or middle school (36.4%). In the elementary school group 39 participants were girls (27.9%) and 50 were boys (35.7%), and in the middle school group 17 were girls (12.1%) and 34 were boys (24.3%).

Regarding academic qualifications, 52.3% of the mothers had a higher education degree, 38.5% had completed a secondary education or an equivalent vocational course, and 9.2% had completed a primary education. Concerning the fathers’ academic studies, 97.6% had higher education degrees, 48.4% had completed secondary education, 24.2% had completed the 3rd cycle of primary education, and 1.6% had completed the 2nd cycle of primary school. 

### 2.2. Instruments

A sociodemographic questionnaire was created to gather information about the parents’ and families’ characteristics (e.g., the age and academic qualifications of the parents).

The Questionnaire of Parental Involvement-Parents’ Version (QPI-PV) [58] was used to assess parental involvement (PI). This 24-item instrument is answered according to a four-point Likert-type scale (from 1 = Not true at all to 4 = Very True) and composed of four subscales: (1) PI in activities at school and volunteering (6 items: e.g., “I give ideas for organizing activities at school”; α = 0.80); (2) PI in learning activities at home (8 items: e.g., “I usually check if my child does his homework”; α = 0.83); (3) School-family communication (6 items: e.g., “When there is any problem with my child at school, I try to inform the teacher”; α = 0.73); (4) Activities at school and parents’ meetings (4 items: e.g., “I go to the parents’ meetings that are scheduled by the teacher”; α = 0.73). The PI subscales’ scores were calculated, inclusive of the total score (α = 0.90), and higher results indicated higher levels of the respective PI. The Cronbach’s alpha coefficient for the total QPI-PV was 0.90 and 0.91 for T1 and T3, respectively.

The Parental Stress Scale (PSS) [59,60] was used. This 18-item instrument assesses the levels of stress experienced by parents according to four dimensions: (1) Parental Concerns (i.e., level of closeness with the child, 5 items: e.g., “It is difficult to balance different responsibilities because of my child[ren]”; α = 0.62); (2) Satisfaction (i.e., level of pleasure in their role as parents, 6 items: e.g., “I feel close to my child(ren)”; α = 0.62); (3) Lack of Control (i.e., negative and positive emotions arising from parenting, 5 items: e.g., “I feel overwhelmed by the responsibility of being a father [mother]”; α = 0.62); and (4) Fears and Anxieties (i.e., difficulties related to parenthood, 2 items: e.g., “Sometimes I wonder if I am doing enough for my child(ren)”; α = 0.62). This instrument is answered using a Likert-type scale, ranging from 1 (Strongly Disagree) to 5 (Strongly Agree). The sum of the scale ranges from 18 to 90, and higher values indicate higher levels of parental stress (α = 0.71). The authors established three categorized intervals, namely low level (18–40), medium level (43–66), and high level (67–90) [60]. The questionnaire showed satisfactory internal reliability indexes in this study, specifically at the first and third times (TI α = 0.71; T3 α = 0.75). In the case of the second time, the reliability index was minimally satisfactory due to the proximity to the minimum reference values (α = 0.62).

The children’s Quality of Life [51,61] was evaluated using the KIDSCREEN-10. This instrument is composed of 10 items that assess QoL (e.g., “Did your child feel sad?”; α = 0.62), and the answers are rated on a five-point Likert-type scale (from 1 = Not at all to 5 = Totally). Higher values indicate a feeling of happiness, perceived adequacy, and satisfaction, while lower values reflect feelings of unhappiness, dissatisfaction, and inadequacy regarding the various contexts of children’s lives.

### 2.3. Procedures 

This study was approved by the Scientific Commission of the Psychology and Sciences Education Department, University of Algarve, Reference No. E-UALG/2020/25976. After obtaining the consent of the original authors for the several instruments used, they were applied using the Google Forms platform. The sociodemographic questionnaire was answered only at T1. The QPI-PV was applied at T1 and T3. The KIDSCREEN-10 and PSS were always applied (i.e., T1, T2, and T3). Data collection was performed with a non-probability convenience sampling procedure using the snowball strategy [62], online, and invitations were sent through social media (e.g., Facebook, Instagram, WhatsApp). Although more parents were expected to participate (i.e., according to G*Power analysis, *n* > 158), due to the study design (i.e., longitudinal, with collections in specific periods), the sample size was the one possible with these conditions.

In T1 (during lockdown, in January of 2021), all participants were informed about the study objective, times, respect for free will without any prejudice in case of a refusal to participate, guarantee of confidentiality of the data provided, and the identities of the researchers who would access it. T2 occurred in March 2021, during the post-lockdown period. Parents were contacted via email to complete the second questionnaire. T3 took place at the end of June of the same year (after lockdown and at the end of the school year), when parents were contacted again via email to complete the final online questionnaire.

### 2.4. Data Analysis

IBM SPSS 29.0.1.0 (IBM Corp., Chicago, IL, USA) was used for data processing. Since we used an online platform with mandatory answers, we had no missing data. Descriptive statistics were applied (i.e., measures of central and non-central tendency as mean, measures of dispersion as amplitude and standard deviation, and asymmetry were considered preferably if they fell into the range [−0.5, 0.5], and treated as kurtosis–mesokurtic when the distribution tended to zero and was in the range [−0.5, 0.5]). Statistical assumptions for parametrical tests, such as the normality of distribution, homogeneity of variance, linearity, and independence, were checked. Alpha Cronbach’s values ranging from 0.70 to 0.80 were considered adequate, above 0.80 very good, and between 0.60 and 0.70 poor but acceptable [63,64]. To test differences among all continuous variables (i.e., PI, children’s QoL), paired-samples Student’s *t*-tests were used between T1 and T2, and to test all three times (and interactions) a repeated measures ANOVA approach was also used. After the verification of the optimal conditions (normality assessed by Shapiro-Wilk, and homogeneity with the Levene Test), the results were interpreted according to a = 5%. When the homogeneity condition was not confirmed, the corrected forms were used (i.e., Welsch test or Brown-Forsyth). Effect size was assessed with the Cohen’s coefficient (small effect *d* = 0.20, medium effect *d* = 0.50, and large effect *d* = 0.80) and Partial *η*^2^ (small effect = 0.01; medium effect = 0.06; and large effect = 0.14). In the end, a power analysis was performed with G*Power 3.1 [65] and analyzed according to the recommended score (1-*β* ≥ 0.80) [66].

To explore the contribution of different dimensions to the child’s QoL, correlations were computed, and the contribution of each predictor and of the total model were determined by using the significative variables in multiple linear regressions (Enter method). The analysis of variance was measured using *R*^2^ and the level of significance was verified using *β* and *p* [64,66].

## 3. Results

### 3.1. Comparison of Childrens’ QoL and Parents’ Stress (T1, T2, T3) 

At the beginning of the lockdown (T1; Table 1), the families indicated a satisfactory average level of children’s QoL (*M* = 3.26; *SD* = 0.46), and higher average scores of satisfaction (*M* = 4.28; *SD* = 0.04), followed by fears and anxieties (*M* = 3.84; *SD* = 0.10), and lack of control (*M* = 3.40; *SD* = 0.04). Parental concerns *(M* = 2.04; *SD* = 0.07) had lower mean levels. 

**Figure 1:**
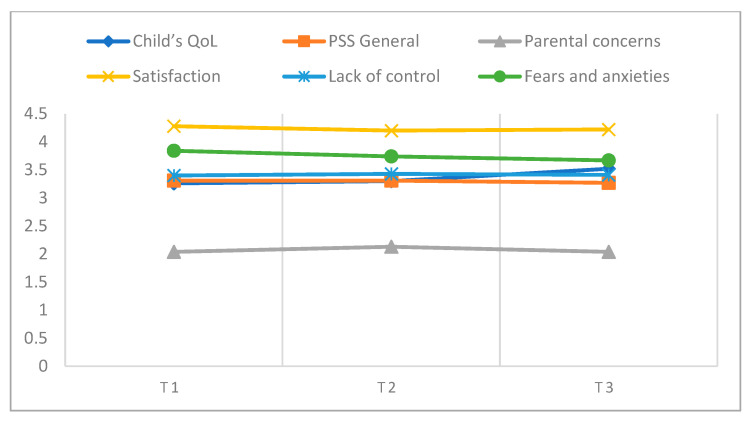
Children QoL and PS mean scores at all times assessed (T1, T2, and T3).

The comparison of the three times (Table 1; Figure 1) reveals significant differences, with a large effect on children’s QoL (*F* (2, 172]) = 15.30; *p* ≤ 0.001; Partial *η*^2^ = 0.15), and marginal differences in parents’ satisfaction levels, with a small effect (*F* (2, 172) = 2.53; *p* = 0.083; Partial *η*^2^ = 0.03). Also, a moderate effect was found in the parents’ general level of stress (*F* (2, 172) = 1.10; *p* = 0.336; Partial *η*^2^ = 0.08), although this effect was not significant. A more refined analysis of the QoL shows significant differences of moderate magnitude between T1 and T2 and T2 and T3 (T1 and T2: *t* (108) = −1.17, *p* = 0.243, *d* = −0.12; T1 and T3: *t* (87) = −5.10, *p* = <0.001, *d* = −0.54; T2 and T3: *t* (86) = −4.21, *p* = <.001, *d* = −0.45), showing a small increase during the three times (T1: *M* = 3.26; *SD* = 0.46; T2: *M* = 3.30; *SD* = 0.47; T3: *M* = 3.52; *SD* = 0.43).

Regarding parental stress (PS) in all its dimensions (i.e., parental concerns [PC], satisfaction in their role as parents [SRP], lack of control [LC], and fears and anxieties [FA]) no significant differences were found between times (Table 1; Figure 1).

### 3.2. Comparison of Parents’ Involvement in T1 and T3 

**Figure 2:**
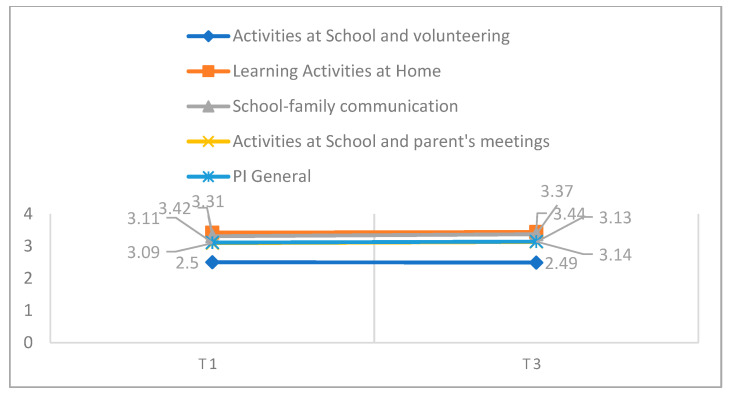
PI mean scores in the times assessed (T1 and T3).

Regarding PI (Table 2; Figure 2), at the beginning of the lockdown period (T1), parents revealed higher levels of learning activities at home (LAH; *M* = 3.42; *SD* = 0.42), while activities at school and volunteering (ASV) had the lower scores (*M* = 2.50; *SD* = 0.62). The PI levels were very similar between the lockdown period and the end of the school year in all dimensions, showing no significant differences (ASV: *t* (87) = 0.10, *p* = 0.920, *d* = 0.01; LAH: *t* (87) = −0.53, *p* = 0.599, *d* = 0.05; SFC: *t* (87) = −1.38, *p* = 0.171, *d* = 0.13; ASPM: *t* (87) = −0.90, *p* = 0.370, *d* = 0.07).

### 3.3. Interaction between Parents’ Level of Education x Times

Regarding the interaction between the parents’ education levels and the three times assessed (Table 3), the results reveal no significant differences in the children’s QoL (*F* (2, 170) = 2.07; *p* = 0.130; Partial *η*^2^ = 0.02), and an almost-significant difference in activities at school and parents’ meetings, with a small-moderate effect (*F* (1, 86) = 3.65; *p* = 0.060; Partial *η*^2^ = 0.04). Parents with a lower education level showed a decrease along T1 and T3 (T1_NotHigher_: *M* = 3.26; *SD* = 0.08; T3_NotHigher_: *M* = 3.22; *SD* = 0.08) and parents with a higher education level presented a rise in the mean scores (T1_NotHigher_: *M* = 2.87; *SD* = 0.09; T3_NotHigher_: *M* = 3.02; *SD* = 0.09). Concerning PS, the results revealed a significant interaction of moderate magnitude in satisfaction (*F* (2, 170) = 4.74; *p* = 0.010; Partial *η*^2^ = 0.05), where T2 had the lowest scores in both groups (T1_NotHigher_: *M* = 4.36; *SD* = 0.05; T2_NotHigher_: *M* = 4.20; *SD* = 0.05; T3_NotHigher_: *M* = 4.22; *SD* = 0.05; T1_Higher_: *M* = 4.17; *SD* = 0.06; T2_Higher_: *M* = 4.22; *SD* = 0.05; T3_Higher_: *M* = 4.21; *SD* = 0.06).

### 3.4. Analysis of the Predictors of the Children’s Quality of Life

To determine the potential predictors of the child’s QoL, a multiple regression analysis by times was carried out (i.e., T1, T2, and T3), considering the type of parent, parent’s education level, parental involvement, and parental stress (Table 4).

Regarding the T1 model, only PI made a significant contribution to explaining the child’s QoL (*β* = 0.20; *p* = 0.032) and all the domains explained just 6%. In the T2 model, the three considered predictors explained 2% of the variance in the child’s QoL and none of the domains was significant. It should be noted that the analysis of parental involvement predictors was not carried out at the second time, as this data was only collected at the first and third times of the study.

The tested model at T3 shows the PI as the predictor of the children’s QoL (*β* = 0.34; *p* = 0.002). The four predictors considered explain 12% of the variance (*p* = 0.029).

## 4. Discussion

Considering the challenges and adversities that parents can sometimes face, especially during specific situations of vulnerability such as the one that occurs during COVID-19 lockdowns, we intended to analyze its impact on parental involvement, stress, and children’s QoL. Therefore, our RQ1 explored whether PI, PS, and children’s QoL were affected by a lockdown (T1, T2, T3). Although our H_1_ considered an increase, PI and PS did not show the expected change over the three times assessed. The results show that, generally, in T1 (i.e., before the lockdown), parents assess PI and children’s QoL at medium levels, marking the PS satisfaction at the higher level, which relates to their satisfaction with their roles as parents. This is congruent with several reported studies [56,67], especially a study carried out by Demaria and Vicary [68]. This study indicated that many parents reported a positive impact of the lockdown on their relationships with their children, strengthening family interactions. Since most of the participants were part of stable households from a psychosocial and economic point of view, the positive assessments made regarding the children’s QoL may reflect this stability.

On the other hand, parents reported low levels of parental concerns (that is, preoccupations regarding the closeness with the child), which could be related to the strong modifications that parents felt during the pandemic, since some caregivers had to begin to work from home, experiencing workload changes and dealing with children’ schoolwork and learning time [67,69].

The comparison between the three times (i.e., before, after, and with no lockdown) revealed differences in children’s QoL, namely an increase. These findings invalidate our H_2_. Most of the research developed during the pandemic period did not find similar results (e.g., [70,71,72,73,74,75,76,77]), reporting a decrease in children’s QoL and mental health. Others found differently and pointed out that some stability or maintenance in children’s QoL [56], could be the result of several causes, including some aspects of the children’s lives that did not change [77], including engaging in a healthy lifestyle [78]. Considering that we did not have access to most of these investigations when they were occurring, we stated our hypotheses according to the available research.

The level of parental satisfaction also shows an almost-significant difference, since participants reported a slight decrease in their assessments. This is in line with studies that highlight several stressors that affect parenting and parent-child relations, such as digital access to education, balancing parental workloads with children’s learning, and managing the educational needs of multiple children [79]. However, other studies in Portugal suggest a different impact on parental domain stress [80,81].

Although we expected otherwise, no differences were found in PI at the two times it was assessed (i.e., T1 and T3). These findings can be attributed to the transition from in-person learning to online learning, which resulted in increased parental commitment and dedication [39,79,82], and closer collaboration between teachers and parents, as they were more concerned with their children’s engagement level and learning outcomes [40]. These trends align with similar research conducted during COVID-19 lockdowns [83,84]. The pandemic period and its consequent lockdown were also considered as a period of positive family moments, eliciting memorable experiences, and thereby fostering family resilience [85].

Even though parents whose children are struggling academically may find less satisfaction in their parenting role and may also experience heightened stress [86], these results can be justified with reference to the children’s age (between 6 and 12 years old) considering that studies reveal PI can change over children’s development [3,17]. According to Boonk et al. [3], as a child grows older, the PI in school activities decreases and other forms of support become more relevant, such as expectations regarding academic success, providing opportunities to explore and boost the child’s motivations, and more advising on relevant academic subjects [17]. Beyond that, as Castro and colleagues [1] showed, primary school is one level of education where the relationship between PI and academic results is stronger. Additionally, most European children and adolescents have very high levels of access to the Internet, both within and outside their homes [87], so it was easy for them to adapt to online learning.

Moving on to our RQ2, it is possible to conclude that engagement in school activities and parent meetings increases in the parents with superior education levels and decreases in the parents with lower education levels, which confirms our H_3_. Our results are in line with the research conducted by Shao et al. [88], in which the parents’ level of education had a negative moderating effect on parental involvement and satisfaction, indicating that parents with higher levels of education are less involved in their children’s tutoring compared to parents with lower levels of education. Or, on the other hand, more educated parents can provide greater support for their children’s autonomy. Nonetheless, the research shows different results when analyzing these variables, as other studies show no significant links between parents’ level of education and parental involvement in children’s education [89], or find that parents with a higher level of education consider themselves more competent to help their children with schoolwork and their children to be more competent in school subjects than do parents with lower levels of education [90].

Additionally, another difference observed was that PS satisfaction decreased in the non-superior education group, but slightly increased in the superior education group. We can hypothesize that this oscillation in the lack of satisfaction with the parental role of these groups may be related to financial issues, if we consider that the parents’ educational qualifications are related to the financial income of the households. This result is congruent with others and shows that a parent’s level of education had a negative moderating effect on parental involvement and satisfaction, indicating that parents with higher levels of education are less involved in their children’s tutoring compared to parents with lower levels of education [88].

Concerning the RQ3, the analysis of the predictive role of the child’s QoL shows that the PI contributes significantly at both times, which fully supports our H_4_. These results were expected and based on the period experienced and the predominance of stable and participative households in the children’s lives. As to the positive relationship established between IVPs and children’s QoL, it is probable that such involvement acts as a protective factor that allows families to thrive in the face of adversity. This is particularly relevant in the context of a lockdown, when normal routines and support systems are disrupted [85,91], emphasizing the importance of parental involvement in relieving the challenges posed during these periods.

Despite the scarcity of literature about QoL and PI, a positive correlation has been found with life satisfaction, measured as a subjective evaluation of the QoL of children [92,93]. The studies mainly focus on the relationship between children’s life satisfaction and the overall perceived QoL of their family, neighborhood, school, and peer relationships [94]. QoL cannot be attributed only to the absence of problems, but must incorporate degrees of positive experience, such as happiness, satisfaction, and meaning to reflect the full range of well-being [95], and PI is an unavoidable indicator of a child’s well-being. Therefore, parents’ involvement in their children’s daily life routines and experiences can constitute a very crucial protective factor for the children’s QoL and well-being [93] and a preventive mechanism against ill-being [96,97].

## 5. Limitations

The present study has limitations that should be considered, including the times of the study, the selection of families, and sample size. Considering the design (longitudinal, three times) and the specific and special conditions that were lived during the COVID-19 lockdowns, the adoption of other strategies for data collection was not possible. Other studies also reported these difficulties (e.g., [40]). One consequence was that parents who participated in our study had a higher level of education than can be expected for the Portuguese population in general. This was a predictable phenomenon, since the data collection was done via an online platform. In-person data collection could help to adjust the sample size [67]. Although the sample size was not the one initially planned, it was the possible one given the conditions. It would have been relevant to include children as a source of data and compare the responses of parents and children included in the households, allowing an analysis of the implications and practical benefits of involvement in children’s QoL. Additionally, it would have been valuable to track parents’ employment status during the lockdown. Mixed methods could also be used to elicit more information and insight from the participants. Also, some instruments revealed constraints and inadequacy of the items to the realities of the families and their relationships with the school context. Nevertheless, it is important to highlight that we had to use the available instruments (i.e., the QPI-PV) and choose the easier to answer (e.g., Kidscreen-10). We believe that other versions could be used (e.g., Kidscreen-27) and other instruments could be selected (e.g., with respect to parental involvement) [77]. Although the period of the COVID-19 pandemic has been circumscribed, future opportunities should contemplate enlarging parental sampling, considering other SES and education levels, and other variables such as school achievement and other actors’ perceptions (e.g., children and teachers).

## 6. Conclusions

The significant contribution of the present study can be its highlighting of the fact that, even with the fear-inducing impact of the COVID-19 pandemic on the entire population, it did not impose severe constraints on all families, regardless of social status or cultural/racial background. Furthermore, the effects were not persistent and did not significantly disrupt the dynamics within the analyzed sample. Despite the challenges, parents demonstrated the ability and competence to uphold their children’s QoL and support their educational activities, adapting to the unique circumstances of this historical period.

The process of parenthood presumes the satisfaction of the child’s physical, emotional, cognitive, and social needs, with the aim for children to become more autonomous [3,98]. Since children’s QoL is strongly related to their mental health and subjective well-being, the protective nature of the partnership between the family, school, and community context is a key point [57], especially during a pandemic. More than two years after the end of the COVID-19 pandemic, we still do not fully understand how exposure to the pandemic has affected mental health outcomes in the general population, especially in children [99]. In fact, the school day provides a supportive and stimulating environment that may protect children and adolescents from behaviors that are adverse to health and well-being [99,100]. The present study emphasizes that, despite the difficult and uncertain period we lived through during the pandemic, these parents were able to maintain important aspects of their children’s lives. These include academic adjustment, achieved through parental involvement, and quality of life, which was managed effectively despite the stress during this period.

## Figures and Tables

**Table 1:** Comparison of the dimensions assessed between the three times (*n* = 88).

Dimensions	T1 *M* (*SD*)	T2 *M* (*SD*)	T3 *M* (*SD*)	*F*	*p*	Partial *η*^2^
Child’s QoL	3.26 (0.46)	3.30 (0.47)	3.52 (0.43)	15.30	<0.001	0.15
PSS General	3.31 (0.04)	3.31 (0.03)	3.27 (0.04)	0.68	0.509	0.08
Parental concerns	2.04 (0.07)	2.13 (0.07)	2.04 (0.07)	1.10	0.336	0.01
Satisfaction	4.28 (0.04)	4.20 (0.03)	4.22 (0.04)	2.53	0.083	0.03
Lack of control	3.40 (0.04)	3.43 (0.03)	3.41 (0.04)	0.25	0.783	0.00
Fears and anxieties	3.84 (0.10)	3.74 (0.09)	3.67 (0.10)	1.24	0.292	0.01

Note. T1 = Time 1; T2 = Time 2; T3 = Time 3; PSS General = Parent’s general level of Stress; *M* = Mean; *SD* = Standard Deviation; *F =* Statistical test *Z*; *p* = *p*-value; *η*^2^ = Eta partial square (Effect size).

**Table 2:** Comparison of the dimensions of PI in T1 and T3 (*n* = 88).

Dimensions	T1	T3	*t*	*p*	*d*
	*M* (*SD*)	*M* (*SD*)			
ASV—Activities at School and Volunteering	2.50 (0.62)	2.49 (0.65)	0.10	0.920	0.01
LAH—Learning Activities at Home	3.42 (0.42)	3.44 (0.43)	−0.53	0.599	0.05
SFC—School-family Communication	3.31 (0.44)	3.37 (0.46)	−1.38	0.171	0.13
ASPM—Activities at School and parent’s meetings	3.09 (0.61)	3.13 (0.57)	−0.90	0.370	0.07
PI General	3.11 (0.40)	3.14 (0.41)	−0.78	0.438	−0.08

Note. T1 = Time 1; T2 = Time 2; T3 = Time 3; *M* = Mean; *SD* = standard deviation; *t =* statistical test; *p* = *p*-value; *d =* d Cohen’s d (Effect size).

**Table 3:** Descriptive statistics and interaction between times and parents’ level of education (*n* = 87).

Dimensions	Not Superior (*n* = 50)			Superior (*n* = 37)			*F*	*p*	*η*^2^ Partial
	T_1_ (*SD*)	T_2_ (*SD*)	T_3_ (*SD*)	T_1_ (*SD*)	T_2_ (*SD*)	T_3_ (*SD*)			
Child’s QoL	3.23 (0.07)	3.36 (0.07)	3.51 (0.06)	3.30 (0.08)	3.23 (0.08)	3.54 (0.07)	2.07	0.130	0.02
PI Activities at School and Volunteering	2.54 (0.08)	(a)	2.49 (0.09)	2.44 (0.10)	(a)	2.49 (0.11)	0.69	0.409	0.01
PI Learning Activities at Home	3.52 (0.06)	(a)	3.52 (0.06)	3.29 (0.07)	(a)	3.34 (0.07)	0.28	0.596	0.00
PI School-family Communication	3.38 (0.06)	(a)	3.44 (0.07)	3.23 (0.07)	(a)	3.28 (0.08)	0.01	0.930	0.00
PI Activities at School and parent’s meetings	3.26 (0.08)	(a)	3.22 (0.08)	2.87 (0.09)	(a)	3.02 (0.09)	3.64	0.060	0.04
PI General	3.20 (0.06)	(a)	3.19 (0.06)	3.00 (0.06)	(a)	3.06 (0.07)	0.90	0.346	0.01
PS Parental concerns	2.14 (0.09)	2.21 (0.09)	2.07 (0.10)	1.91 (0.10)	2.02 (0.11)	2.00 (0.10)	0.69	0.501	0.01
PS Satisfaction	4.36 (0.05)	4.20 (0.05)	4.22 (0.05)	4.17 (0.06)	4.22 (0.05)	4.21 (0.06)	4.73	0.010	0.05
PS Lack of control	3.48 (0.05)	3.47 (0.04)	3.42 (0.05)	3.29 (0.06)	3.36 (0.05)	3.39 (0.06)	1.61	0.202	0.02
PS Fears and anxieties	3.80 (0.13)	3.61 (0.12)	3.64 (0.13)	3.89 (0.16)	3.92 (0.14)	3.70 (0.15)	0.73	0.483	0.01
PS General	3.38 (0.05)	3.33 (0.04)	3.29 (0.05)	3.21 (0.06)	3.28 (0.05)	3.26 (0.06)	2.27	0.107	0.03

Note. PI = parental Involvement; PS = Parental Stress; *M* = Mean; *SD* = Standard Deviation; *F* = Statistical test; *p = p*-value; *η*^2^ partial = Partial Eta-Squared (Effect size); (a) Variable not assessed in this period.

**Table 4:** Predictors of child QoL.

Children QoL	T1 (*n* = 129)	T2 (*n* = 110)	T3 (*n* = 88)
	*β*	*β*	*β*
Father/mother	0.01	−0.00	−0.14
Parents’ education level	0.08	−0.12	0.14
PI	0.20 *	(a)	0.34 **
PS	−0.12	-0.10	-0.01
R^2^	0.06	0.02	0.12 *

Note. (a) = not assessed; * *p* < 0.05; ** p < 0.01; PI = Parental Involvement; PS = Parental Stress; *R*^2^ = Proportion of variance; *n* = Sample; *β =* Regression standardized coefficients.

## Data Availability

The data can be made available for consultation from the corresponding author upon request due to restrictions of privacy.

## References

[1] Castro M., Expósito-Casas E., López-Martín E., Lizasoain L., Navarro-Asencio E., Gaviria J.L. (2015). Parental involvement on student academic achievement: A meta-analysis. Educ. Res. Rev..

[2] Barger M.M., Kim E.M., Kuncel N.R., Pomerantz E.M. (2019). The relation between parents’ involvement in children’s schooling and children’s adjustment: A meta-analysis. Psychol. Bull..

[3] Boonk L., Gijselaers H.J.M., Ritzen H., Brand-Gruwel S. (2018). A review of the relationship between parental involvement indicators and academic achievement. Educ. Res. Rev..

[4] Epstein J.L., Sanders M.G., Bornstein M.H. (2002). Family, school, and community partnerships. Handbook of Parenting: Volume 5: Practical Issues in Parenting.

[5] Epstein J.L., Sanders M.G., Simon B.S., Salinas K.C., Jansorn N.R., Van Voorhis F.L. (2002). Family, School, and Community Partnerships: Your Handbook for Action.

[6] Hill N.E., Chao R.K. (2009). Families, Schools, and the Adolescent: Connecting Research, Policy, and Practice.

[7] Szumski G., Karwowski M. (2017). Parents’ engagement in the education of lower secondary school students with and without special educational needs—Which strategies bring expected results?. Eduk. Q..

[8] Jeynes W. (2005). A meta-analysis of the relation of parental involvement to urban elementary school student academic achievement. Urban Educ..

[9] LaRocque M., Kleiman I., Darling S.M. (2011). Parental involvement: The missing link in school achievement. Prev. Sch. Fail..

[10] Hornby G. (2011). Parental Involvement in Childhood Education: Building Effective School-Family Partnerships.

[11] Oswald D.P., Zaidi H.B., Cheatham D.S., Brody K.G. (2018). Correlates of parent involvement in students’ learning: Examination of a national data set. J. Child. Fam. Stud..

[12] Guedes F., Cerqueira A., Gaspar S., Gaspar T., Moreno C., Matos M. (2022). Family Environment and Portuguese Adolescents: Impact on Quality of Life and Well-Being. Children.

[13] Hornby G., Blackwell I. (2018). Barriers to parental involvement in education: An update. Educ. Rev..

[14] Steinberg L., Lamborn S.D., Dornbusch S.M., Darling N. (1992). Impact of parenting practices on adolescent achievement: Authoritative parenting, school involvement, and encouragement to succeed. Child Dev..

[15] Desforges C., Abouchaar A. (2003). The Impact of Parental Involvement, Parental Support and Family Education on Pupil Achievements and Adjustment: A Literature Review.

[16] DePlanty J., Coulter-Kern R., Duchane K.A. (2007). Perceptions of parent involvement in academic achievement. J. Educ. Res..

[17] Choi N., Chang M., Kim S. (2015). A Structural Model of Parent Involvement with Demographic and Academic Variables. Psych. Sch..

[18] Cen S., Aytac B. (2017). Ecocultural perspective in learning disability: Family support resources, values, child problem behaviors. Learn. Disabil. Q..

[19] Gurung A., Dorji K., Nepal A. (2020). Parental Involvement in Students’ Academic Performance: A Study Based at Pelrithang Middle Secondary School. J. Community Dev. Res..

[20] Krenz A. (2010). La Distinction Reloaded: Returns to Education, Family Background, Cultural and Social Capital in Germany.

[21] Pleck J.H., Masciadrelli B.P., Lamb M.E. (2004). Paternal involvement by U.S. residential fathers: Levels, sources, and consequences. The Role of the Father in Child Development.

[22] Pleck J.H. (2007). Why could father involvement benefit children?. Appl. Dev. Sci..

[23] Justo A.P., Lipp M.E.N. (2010). A influência do estilo parental no stress do adolescente. Bol. Acad. Paul. Psicol..

[24] Pires M., Hipólito J., Jesus S.N. Estilos Parentais e Stress Infantil. Proceedings of the Actas I Congresso Luso-Brasileiro de Psicologia da Saúde—Simpósio: Vulnerabilidade e Desenvolvimento.

[25] López M.J.R., Chavez M.L.M., Quintana J.C.M. (2010). Parentalidad Positiva y Políticas Locales de Apoyo a Las Famílias.

[26] Pérez-Padilla J., Ayala-Nunes L., Hidalgo M.V., Nunes C., Lemos I., Menéndez S. (2017). Parenting and stress: A study with Spanish and Portuguese at-risk families. Int. Soc. Work.

[27] Ward K.P., Lee S.J. (2020). Mothers’ and fathers’ parenting stress, responsiveness, and child wellbeing among low-income families. Child. Youth Serv. Rev..

[28] Maat D.A., Jansen P.W., Prinzie P., Keizer R., Franken I.H., Lucassen N. (2021). Examining longitudinal relations between mothers’ and fathers’ parenting stress, parenting behaviors, and adolescents’ behavior problems. J. Child Fam. Stud..

[29] Morris E., Farrell A. (2020). Delivering Distance Learning in Emergencies: A Review of Evidence and Best Practice.

[30] Al-Garadi M.A., Yang Y.C., Sarker A. (2022). The Role of Natural Language Processing during the COVID-19 Pandemic: Health Applications, Opportunities, and Challenges. Healthcare.

[31] Tso W.W., Wong R.S., Tung K.T., Rao N., Fu K.W., Yam J.C., Wong I.C. (2022). Vulnerability and resilience in children during the COVID-19 pandemic. Eur. Child Adolesc. Psychiatry.

[32] Wang G., Zhang Y., Zhao J., Zhang J., Jiang F. (2020). Mitigate the effects of home confinement on children during the COVID-19 outbreak. Lancet.

[33] Achterberg M., Dobbelaar S., Boer O.D., Crone E.A. (2021). Perceived stress as mediator for longitudinal effects of the COVID-19 lockdown on wellbeing of parents and children. Sci. Rep..

[34] Panchal U., Salazar de Pablo G., Franco M., Moreno C., Parellada M., Arango C., Fusar-Poli P. (2023). The impact of COVID-19 lockdown on child and adolescent mental health: Systematic review. Eur. Child Adolesc. Psychiatry.

[35] Ghosh R., Dubey M.J., Chatterjee S., Dubey S. (2020). Impact of COVID-19 on children: Special focus on the psychosocial aspect. Minerva Pediatr..

[36] Magalhães L., Nascimento C., Antunes A.P., Martins S., Yunes M.A.M., Almeida A. (2021). Perceção de experiências marcantes do confinamento em famílias portuguesas e brasileiras: Um estudo qualitativo. New Trends Qual. Res..

[37] Ordem dos Psicólogos Portugueses (2020). COVID-19: Como Manter Atividades de Ensino, Aprendizagem e Formação à Distância.

[38] Novianti R., Puspitasari E., Maria I. (2021). Parents’ Involvement in Children’s Learning Activities During The COVID-19 Pandemic. J. PAJAR Pendidik. Dan Pengajaran.

[39] Knopik T., Blaszczak A., Maksymiuk R., Oszwa U. (2021). Parental Involvement in Remote Learning during the COVID-19 Pandemic-Dominant Approaches and Their Diverse Implications. Eur. J. Educ..

[40] Suárez N., Fernández E., Regueiro B., Rosário P., Xu J., Núñez J.C. (2022). Parental Involvement in Homework During Covid-19 Confinement. Psicothema.

[41] Van Ballegooijen H., Goossens L., Bruin R.H., Michels R., Krol M. (2021). Concerns, quality of life, access to care and productivity of the general population during the first 8 weeks of the coronavirus lockdown in Belgium and the Netherlands. BMC Health Serv. Res..

[42] Cusinato M., Iannattone S., Spoto A., Poli M., Moretti C., Gatta M., Miscioscia M. (2020). Stress, Resilience, and Well-Being in Italian Children and Their Parents during the COVID-19 Pandemic. Int. J. Environ. Res. Public Health.

[43] Lawrence K.C., Fakuade O.V. (2021). Parental involvement, learning participation and online learning commitment of adolescent learners during the COVID-19 lockdown. Res. Learn. Technol..

[44] Panaoura R. (2020). Parental Involvement in Children’s Mathematics Learning Before and During the Period of the COVID-19. Soc. Educ. Res..

[45] Sari D.K., Maningtyas R.T. Parents’ involvement in distance learning during the COVID-19 pandemic. Proceedings of the 2nd Early Childhood and Primary Childhood Education.

[46] Spinelli M., Lionetti F., Setti A., Fasolo M. (2021). Parenting Stress During the COVID-19 Outbreak: Socioeconomic and Environmental Risk Factors and Implications for Children Emotion Regulation. Fam. Process..

[47] Skaliotis E. (2010). Changes in parental involvement in secondary education: An exploration study using the longitudinal study of young people in England. Br. Educ. Res. J..

[48] Drummond K.V., Stipek D. (2004). Low-Income Parents’ Beliefs about Their Role in Children’s Academic Learning. Elem. Sch. J..

[49] Davis K.M., Lambie G.W. (2005). Family Engagement: A Collaborative, Systemic Approach for Middle School Counselors. Prof. Sch. Couns..

[50] Golubović Š., Škrbić R. (2013). Agreement in quality of life assessment between adolescents with intellectual disability and their parents. Res. Dev. Disabil..

[51] Gaspar T., Matos M.G. (2008). Qualidade de Vida em Crianças e Adolescentes: Versão Portuguesa dos Instrumentos KIDSCREEN-52: Aventura Social e Saúde.

[52] Nobari H., Fashi M., Eskandari A., Villafaina S., Murillo-Garcia A., Pérez-Gómez J. (2021). Effect of COVID-19 on Health-Related Quality of Life in Adolescents and Children: A Systematic Review. Int. J. Environ. Res. Public Health.

[53] Wunsch K., Nigg C., Niessner C., Schmidt S.C.E., Oriwol D., Hanssen-Doose A., Burchartz A., Eichsteller A., Kolb S., Worth A. (2021). The Impact of COVID-19 on the Interrelation of Physical Activity, Screen Time and Health-Related Quality of Life in Children and Adolescents in Germany: Results of the Motorik-Modul Study. Children.

[54] Yang Y., Li S., Cai Y., Zhang Q., Ge P., Shang S., Han H. (2023). Effectiveness of telehealth-based exercise interventions on pain, physical function and quality of life in patients with knee osteoarthritis: A meta-analysis. J. Clin. Nurs..

[55] Ravens-Sieberer U., Kaman A., Otto C., Adedeji A., Devine J., Erhart M., Hurrelmann K. (2020). Mental health and quality of life in children and adolescents during the COVID-19 pandemic—Results of the COPSY study. Dtsch. Ärztebl. Int..

[56] López-Aymes G., Valadez M.D.L.D., Rodríguez-Naveiras E., Castellanos-Simons D., Aguirre T., Borges Á. (2021). A mixed methods research study of parental perception of physical activity and quality of life of children under home lock down in the COVID-19 pandemic. Front. Psychol..

[57] Gaspar T., Matos M., Ribeiro J., Leal I. (2008). Promoção de Qualidade de Vida em Crianças e Adolescentes. Psicol. Saúde Doenças.

[58] Pereira A.I.F., Canavarro J.M., Cardoso M.F., Mendonça D.V. (2008). Envolvimento parental na escola e ajustamento em crianças do 1º ciclo do ensino básico. Rev. Port. Pedagog..

[59] Berry J.O., Jones W.H. (1995). The parental stress scale: Initial psychometric evidence. J. Soc. Pers. Relatsh..

[60] Mixão M.L., Leal I., Maroco J., Leal I., Marôco J. (2010). Escala de stress parental. Avaliação em Sexualidade e parentalidade.

[61] Gaspar T., Matos M.G., Pais R., José L., Leal I., Ferreira A. (2009). Health-Related Quality of Life in Children and Adolescents and Associated Factors. J. Cogn. Behav. Psychother..

[62] Ribeiro J.L. (2010). Metodologia de Investigação em Psicologia e Saúde.

[63] Freire T., Almeida L. (2008). Metodologia da Investigação em Psicologia e Educação.

[64] Field A. (2009). Discovering Statistics Using IBM SPSS Statistics, 5th ed.

[65] Faul F., Erdfelder E., Lang A.G., Buchner A. (2007). G* Power 3: A flexible statistical power analysis program for the social, behavioral, and biomedical sciences. Behav. Res. Methods.

[66] Cashen L.H., Geiger S.W. (2004). Statistical Power and the Testing of Null Hypotheses: A Review of Contemporary Management Research and Recommendations for Future Studies. Org. Res. Methods.

[67] Bourion-Bédès S., Rousseau H., Batt M., Tarquinio P., Lebreuilly R., Sorsana C., Legrand K., Tarquinio C., Baumann C. (2022). The effects of living and learning conditions on the health-related quality of life of children and adolescents during the COVID-19 lockdown in the French Grand Est region. BMC Public Health.

[68] Demaria F., Vicari S. (2021). COVID-19 quarantine: Psychological impact and support for children and parents. Ital. J. Pediatr..

[69] Prime H., Wade M., Browne D.T. (2020). Risk and resilience in family well-being during the COVID-19 pandemic. Am. Psychol..

[70] Borualogo I.S., Casas F. (2021). Subjective well-being of bullied children in Indonesia. Appl. Res. Qual. Life.

[71] Epifanio I., Ferrando L., Martínez-García M. Mainstreaming gender in mathematics university teaching and an assessment from students and teachers. Proceedings of the 2021 XI International Conference on Virtual Campus (JICV).

[72] Luijten M.A., van Muilekom M.M., Teela L., Polderman T.J., Terwee C.B., Zijlmans J., Klaufus L., Popma A., Oostrom K.J., van Oers H.A. (2021). The impact of lockdown during the COVID-19 pandemic on mental and social health of children and adolescents. Qual. Life Res..

[73] Mantovani S., Bove C., Ferri P., Manzoni P., Cesa Bianchi A., Picca M. (2021). Children ‘under lockdown’: Voices, experiences, and resources during and after the COVID-19 emergency. Insights from a survey with children and families in the 47 Lombardy region of Italy. Eur. Early Child. Educ. Res. J..

[74] Pizarro-Ruiz J.P., Ordóñez-Camblor N. (2021). Effects of COVID-19 confinement on the mental health of children and adolescents in Spain. Sci. Rep..

[75] Ravens-Sieberer U., Kaman A., Erhart M., Devine J., Schlack R., Otto C. (2022). Impact of the COVID-19 pandemic on quality of life and mental health in children and adolescents in Germany. Eur. Child Adolesc. Psychiatry.

[76] Rossi L., Behme N., Breuer C. (2021). Physical activity of children and adolescents during the COVID-19 pandemic—A scoping review. Int. J. Environ. Res. Public Health.

[77] Vallejo-Slocker L., Sanz J., García-Vera M.P., Fresneda J., Vallejo M.A. (2022). Mental health, quality of life and coping strategies in vulnerable children during the COVID-19 pandemic. Psicothema.

[78] Adıbelli D., Sümen A. (2020). The effect of the coronavirus (COVID-19) pandemic on health-related quality of life in children. Child Youth Serv. Rev..

[79] O’Connor Bones U., Bates J., Finlay J., Campbell A. (2022). Parental Involvement during COVID-19: Experiences from the Special School. Eur. J. Spec. Needs Educ..

[80] Aguiar J., Matias M., Braz A.C., César F., Coimbra S., Gaspar M.F., Fontaine A.M. (2021). Parental Burnout and the COVID-19 Pandemic: How Portuguese Parents Experienced Lockdown Measures. Fam. Relat..

[81] Santos M., Ferraz A., Bernardo A.C., Machado A.M., Evangelista M., Ribeiro I., Pereira M.G. (2023). Family Functioning in a Portuguese Sample of Adults during COVID-19: Does Hope Matter?. Clin. Salud.

[82] Fitriani S., Sari Y.Y. (2023). Through parents’ eyes: Exploring parental involvement’s experiences on online learning. Int. J. Eval. Res. Educ..

[83] Bubb S., Jones M.A. (2020). Learning from the COVID-19 Home-Schooling Experience: Listening to Pupils, Parents/Carers and Teachers. Improv. Sch..

[84] Pek L.S., Mee R.W.M. (2020). Parental involvement on childs education at home during school lockdown. J. Humanit. Soc. Stud..

[85] Antunes A.P., Martins S., Almeida A.T. (2023). Factors Associated with Parenting Adaptability in Facing the First COVID-19 Lockdown: A Study on Portuguese Parents. Healthcare.

[86] Ayala-Nunes L., Jiménez L., Jesus S., Nunes C., Hidalgo V. (2018). An ecological model of well-being in child welfare referred children. Soc. Indic. Res..

[87] Willems R.A., Smith P.K., Culbert C., Purdy N., Hamilton J., Völlink T., Scheithauer H., Fiedler N., Brighi A., Menin D. (2023). Internet Use and Perceived Parental Involvement among Adolescents from Lower Socioeconomic Groups in Europe: An Exploration. Children.

[88] Shao M., He W., Zhao L., Su Y.S. (2022). The Influence of Parental Involvement on Parent Satisfaction: The Moderating Effect of Parental Educational Level and the Number of Children. Front. Psychol..

[89] Vellymalay S.K.N. (2010). Parental involvement in children’s education: Does parents’ education level really matters. Eur. J. Soc. Sci..

[90] Baten E., Vlaeminck F., Mués M., Valcke M., Desoete A., Warreyn P. (2023). The Impact of School Strategies and the Home Environment on Home Learning Experiences During the COVID-19 Pandemic in Children with and Without Developmental Disorders. J. Autism Dev. Disord..

[91] Antunes A.P., Martins S., Magalhães L., Almeida A.T. (2021). Parenting during the COVID-19 Lockdown in Portugal: Changes in Daily Routines, Co-Parenting Relationships, Emotional Experiences, and Support Networks. Children.

[92] Diener E., Diener M., Diener E. (2009). Cross-Cultural Correlates of Life Satisfaction and Self-Esteem. Culture and Well-Being: Social Indicators Research Series.

[93] Nunes C., Martins C., Brás M., Carmo C., Gonçalves A., Pina A. (2022). Impact of an online parenting support programme on children’s quality of life. Children.

[94] Liu G., Zhao Z., Li B., Pan Y., Cheng G. (2023). Parental psychological well-being and parental emotional warmth as mediators of the relationship between family socioeconomic status and children’s life satisfaction. Curr. Psychol..

[95] Wallander J.L., Koot H.M. (2016). Quality of life in children: A critical examination of concepts, approaches, issues, and future directions. Clin. Psychol. Rev..

[96] Brás M., Carmo C., Jesus S. (2017). Estudo das propriedades psicométricas do Inventário de Reconhecimento de Sinais de Alerta para Atos Suicidas. Rev. Iberoam. Diagnóstico Evaluación—E Avaliação Psicol..

[97] Lemos I., Brás M., Lemos M., Nunes C. (2022). Psychological Distress Symptoms and Resilience Assets in Adolescents in Residential Care. Children.

[98] Pereira D.I.F., Alarcão M. (2014). “Parentalidade Minimamente Adequada”: Contributos para a operacionalização do conceito. Anal. Psic..

[99] Renzi A., Conte G., Tambelli R. (2022). Somatic, Emotional and Behavioral Symptomatology in Children during COVID-19 Pandemic: The Role of Children’s and Parents’ Alexithymia. Healthcare.

[100] Eglitis E., Miatke A., Virgara R., Machell A., Olds T., Richardson M., Maher C. (2024). Children’s Health, Wellbeing and Academic Outcomes over the Summer Holidays: A Scoping Review. Children.

